# USING THE MONTREAL COGNITIVE ASSESSMENT IN MILD AGE-RELATED HEARING LOSS

**DOI:** 10.1093/geroni/igae098.3055

**Published:** 2024-12-31

**Authors:** Shraddha Shende, Raksha Mudar

**Affiliations:** Illinois State University, Normal, Illinois, United States; University of Illinois, Champaign, Illinois, United States

## Abstract

Age-related hearing loss (ARHL) is a modifiable risk factor for dementia. There is a need for early detection of cognitive concerns in older adults with ARHL. The Montreal Cognitive Assessment (MoCA) is a well-validated screening tool that has been used to screen cognition in the ARHL population. However, few studies have examined the value of both MoCA total score (MoCA-TS) and MoCA-Memory Index Score (MoCA-MIS) score in ARHL and its relation to complex hearing functions, especially in those with mild hearing loss. We administered MoCA on 15 older adults with mild-to-moderate ARHL and 15 matched normal hearing controls. MoCA-TS and MoCA-MIS were calculated. Participants also completed the Quick Speech-in-Noise (QuickSiN) test and the Hearing Handicap Inventory for Adults (HHIA). Relative to controls, worse scores were obtained in the mild ARHL group on MoCA-TS (p =.031) and MoCA-MIS (p =.039). Bivariate correlations revealed a significant negative association between QuickSiN and MoCA-TS scores (p <.001), with similar trends on MoCA-MIS (p =.051). When the peripheral hearing measure was controlled using partial correlations, the negative correlation between QuickSiN and MoCA-TS remained (p =.004). The correlations of MoCA-TS and MoCA-MIS with HHIA were not significant. Our findings reveal changes in global cognition beyond encoding memory in mild ARHL. Additionally, our findings suggest that complex listening functions assessed using QuickSiN in related to overall performance on MoCA.

